# A Two-Stage Reconstruction Processor for Human Detection in Compressive Sensing CMOS Radar

**DOI:** 10.3390/s18041106

**Published:** 2018-04-05

**Authors:** Kuei-Chi Tsao, Ling Lee, Ta-Shun Chu, Yuan-Hao Huang

**Affiliations:** 1 Department of Electrical Engineering, National Tsing Hua University, Hsinchu 30013, Taiwan; andrew000129@gmail.com (K.-C.T.); tschu@ee.nthu.edu.tw (T.-S.C.); 2 Institute of Communications Engineering, National Tsing Hua University, Hsinchu 30013, Taiwan; leelinpp@gmail.com

**Keywords:** compressive sensing, CMOS radar, ranging

## Abstract

Complementary metal-oxide-semiconductor (CMOS) radar has recently gained much research attraction because small and low-power CMOS devices are very suitable for deploying sensing nodes in a low-power wireless sensing system. This study focuses on the signal processing of a wireless CMOS impulse radar system that can detect humans and objects in the home-care internet-of-things sensing system. The challenges of low-power CMOS radar systems are the weakness of human signals and the high computational complexity of the target detection algorithm. The compressive sensing-based detection algorithm can relax the computational costs by avoiding the utilization of matched filters and reducing the analog-to-digital converter bandwidth requirement. The orthogonal matching pursuit (OMP) is one of the popular signal reconstruction algorithms for compressive sensing radar; however, the complexity is still very high because the high resolution of human respiration leads to high-dimension signal reconstruction. Thus, this paper proposes a two-stage reconstruction algorithm for compressive sensing radar. The proposed algorithm not only has lower complexity than the OMP algorithm by 75% but also achieves better positioning performance than the OMP algorithm especially in noisy environments. This study also designed and implemented the algorithm by using Vertex-7 FPGA chip (Xilinx, San Jose, CA, USA). The proposed reconstruction processor can support the 256×13 real-time radar image display with a throughput of 28.2 frames per second.

## 1. Introduction

The complementary metal-oxide-semiconductor (CMOS) radar system has recently drawn much research attention because of the great demand of low-power actively sensing devices for the internet-of-thing (IOT) system [1,2,3]. The human-centric home-care system is one of the most potential applications of the CMOS radar system, in which an impulse-radio radar transceiver can transmit high-frequency impulses to scan the weak respiration signals and identify the human body in the noisy environments [4,5,6]. However, the high-resolution signal increases the dimension of grid space, leading to the increased computational complexity of the target detection. Thus, the target detection and weak human feature extraction are two important signal processing issues for the human-centric CMOS radar system.

Traditional human detection algorithms detected respiration frequency in either time domain or frequency domain [7,8,9]. However, most of them were developed by using simple sinusoidal respiration model. Our previous work [10] designed a CMOS impulse radar system and developed a respiration feature extraction algorithm for detecting fine human features. The proposed single-input single-output (SISO) impulse radar system can detect single human body in a tunable fixed distance (1D distance) and extract the respiration features based on a four-segment linear waveform model.

The target detection and localization is another signal processing issue for the impulse radar system because of its great computational complexity. Traditional radar systems usually achieve high-accuracy target detection regardless of the computational cost because the radar station is usually equipped with high-performance digital signal processors. For an IOT system, the computational power of the target detection algorithm becomes a critical design issue because of the low-power requirement of wireless sensing nodes. Therefore, many researchers [11,12] applied compressive sensing (CS) reconstruction algorithms to the object detection to eliminate high-power matched filters and high-bandwidth analog-to-digital converters. One the other hand, some researchers designed the shape of the transmitted impulse to satisfy the incoherent constraint of CS. Thus, the reconstruction algorithm can rebuild the target scene with better performance and lower complexity than a classical radar in the literature [13,14].

The reconstruction algorithm dominates the signal processing complexity of a CS radar system [15]. $l_{1}$-minimization (basis pursuit) and greedy reconstruction are two typical algorithms. $l_{1}$-minimization [12,13] has robust reconstruction performance but involves very high complexity. Greedy reconstruction algorithms, such as orthogonal matching pursuit (OMP), has worse performance than $l_{1}$-minimization. Thus, the greedy reconstruction algorithms [16,17,18,19,20,21] are more suitable for low-power hardware implementation. For a certain CS-radar-scanned area, higher resolution leads to higher grid density and higher reconstruction complexity. This design issue is especially stringent for a human-centric wireless sensing system because the respiratory vibration is extremely fine and requires a high resolution radar system. This work proposes a low-complexity two-stage OMP reconstruction algorithm for a single-input multiple-output (SIMO) CS radar. The contributions of the proposed algorithm are twofold. Firstly, the computational complexity of the proposed algorithm is significantly lower than the OMP algorithm, especially when the resolution of the CS radar system increases. Secondly, the detection performance of the proposed algorithm is better than that of the OMP algorithm especially in noisy environments. Finally, a reconstruction processor was designed and implemented for a real-time radar image display for the single-input and multiple-output (SIMO) CS radar system.

The rest of this paper is organized as follows. Section 2 introduces the signal model of the CS radar system and the signal reconstruction algorithms for target detection. Section 3 presents the proposed low-complexity reconstruction algorithm. Section 4 analyses the computational complexity and performance of the proposed algorithm. Section 5 presents the FPGA design and implementation of the proposed reconstruction processor. Finally, conclusions are given in Section 6.

## 2. Compressive Sensing Radar System

### 2.1. Compressive Sensing

The measurement process of compressive sensing system can be expressed by

(1) y=Φr

where y is an M×1 sensing vector; Φ is an M×N measurement matrix; and r is an N×1 vector of the original signal with a higher dimension than y. Signal sparsity and incoherence of sensing matrix are requisites of efficient signal reconstruction [22,23]. r can be described by

(2) r=Ψx

where Ψ is an N×N basis matrix of the sparse domain and x is an N×1 coefficient vector in the sparse domain. There are only a few non-zero elements in x. Using Ψ as basis, signal r can be transformed to the sparse domain as x with only K non-zero elements. K is much smaller than N. Then, the overall compressive sensing signal model can be expressed by

(3) y=Φr=ΦΨx=Ax

where A=ΦΨ is the sensing matrix of the CS system.

### 2.2. SISO Compressive Sensing Radar System

In a single-input single-output compressive sensing impulse radar system, the received signal can be expressed by

(4) $$y\left(t\right)=\sum_{i}p_{i}s\left(t-\tau_{i}\right)$$

where s(t) is transmitted impulse signal. The *i*-th target reflects the transmitted impulse signal with signal gain $p_{i}$ and propagation delay $\tau_{i}$. $d_{i}$ is the distance between the radar and the *i*-th target and can be calculated by $d_{i}=\frac{\tau_{i}c}{2}$, where c is the light velocity. The received signal can be also rewritten as follows:

(5) $$y\left(t\right)=\sum_{i}\alpha_{i}z\left(t;d_{i}\right)$$

where $z(t;d_{i})$ is the echo signal of the *i*-th target and $\alpha_{i}$ is the signal gain coefficient determined by the path loss and other properties of the target, such as energy reflection/absorption ratio of the target.

### 2.3. MIMO Compressive Sensing Radar System

A multiple-input multiple-output (MIMO) radar system has many advantages over a SISO radar system [24]. The MIMO radar can scan targets in a 2-D space by using phase array antennas [25]. On the other hand, multiple transmitted impulse signals can be properly designed to reduce the degree of coherence of the compressive sensing system so as to improve the signal reconstruction accuracy [13,14]. The received signal of the MIMO compressive sensing radar can be expressed by

(6) $$y\left(t\right)=\sum_{k}\alpha_{k}z\left(t;\phi_{k},d_{k}\right)$$

where $\alpha_{k}$ is amplitude coefficient of the *k*-th target, and $\phi_{k}$ and $d_{k}$ are the direction (angle) and distance of the *k*-th target, respectively. The echo signal z(t;ϕ,d) is extended from z(t;d) in Equation (5) with an additional dimension ϕ .

Assume that a MIMO radar system has $N_{R}$ receive antennas and $N_{T}$ transmit antennas, and each receive antenna acquires $N_{S}$ samples. The echo signal matrix of a target with distance *d* and angle ϕ is denoted as Z(t;ϕ,d). Echo signal z(t;ϕ,d) in Equation (6) is a vectorized version of the echo signal matrix Z(t;ϕ,d). Echo signal matrix Z(t;ϕ,d) is an $N_{R}\times N_{S}$ matrix, and echo signal z(t;ϕ,d) is an $N_{R}N_{S}\times 1$ vector. The echo signal matrix Z(t;ϕ,d) can be constructed by using uniformly-spaced linear array (ULA) [12] and can be expressed as follows:

(7) $$Z\left(t;\phi ,d\right)=a_{R}\left(\phi \right)a_{T}^{T}\left(\phi \right)S\left(t-\tau \right)$$

where $a_{R}\left(\phi \right)$ is an $N_{R}\times 1$ receive matrix, $a_{T}\left(\phi \right)$ is an $N_{T}\times 1$ transmit matrix, and S(t-τ) is an $N_{T}\times N_{S}$ transmitted signal matrix. The delay τ can be calculated by using $\tau =\frac{2d}{c}$. In the transmitted signal matrix S(t-τ), each row of the matrix are the delayed transmitted signal $s_{i}\left(t-\tau \right)$ of the *i*-th transmit antenna. Transmit matrix $a_{T}\left(\phi \right)$ and receive matrix $a_{R}\left(\phi \right)$ are given by Equations (9) and (10), respectively, in which $d_{T}$ and $d_{R}$ are the normalized spacings (distance divided by wavelength) of the transmit antennas and receive antennas, respectively [12]. According to Equations (8)–(10), echo signal matrix Z(t;ϕ,d) in Equation (7) can be established. Then, echo signal vector z(t;ϕ,d) in Equation (6) can be generated by vectorizing the echo signal matrix Z(t;ϕ,d).

(8) $$S\left(t-\tau \right)=\left[\begin{array}{ccc} \cdots & N_{s}\;\mathrm{samples}\;\mathrm{of}\;s_{1}\left(t-\tau \right) & \cdots \\ \cdots & N_{s}\;\mathrm{samples}\;\mathrm{of}\;s_{2}\left(t-\tau \right) & \cdots \\ & \vdots & \\ \cdots & N_{s}\;\mathrm{samples}\;\mathrm{of}\;s_{N_{T}}\left(t-\tau \right) & \cdots \end{array}\right]$$

(9) $$a_{T}\left(\phi \right)={\left[\begin{array}{cccc} 1 & e^{j2\pi d_{T}\sin\left(\phi \right)} & \cdots & e^{j2\pi d_{T}\sin\left(\phi \right)\left(N_{T}-1\right)} \end{array}\right]}^{T}$$

(10) $$a_{R}\left(\phi \right)={\left[\begin{array}{cccc} 1 & e^{j2\pi d_{R}\sin\left(\phi \right)} & \cdots & e^{j2\pi d_{R}\sin\left(\phi \right)\left(N_{R}-1\right)} \end{array}\right]}^{T}$$

The target scene of an MIMO radar system is a discrete $N_{r}\;\times \;N_{\theta }$ range-azimuth grid.

**Figure 1:**
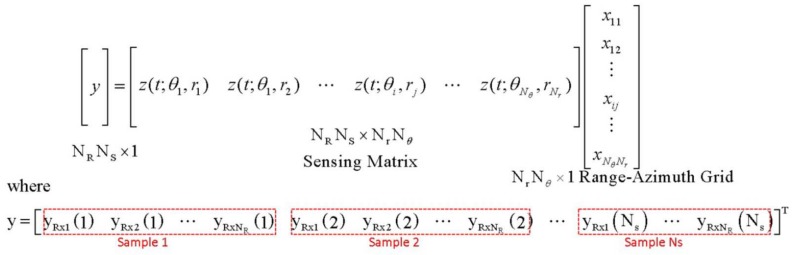
Multiple-input multiple-output (MIMO) compressive sensing radar model.

When a target is at the range-azimuth grid point $\left(\theta_{i},r_{j}\right)$ within the coverage of the MIMO radar system, that is, $1\leq i\leq N_{\theta }$ and $1\leq j\leq N_{r}$, the received signal can be expressed by

(11) $$y\left(t\right)=\sum_{i=1}^{N_{\theta }}\sum_{j=1}^{N_{r}}x_{ij}z\left(t;\theta_{i},r_{j}\right),$$

where $z(t;\theta_{i},r_{j})$ is the echo signal of the target at $\left(\theta_{i},r_{j}\right)$ and $x_{ij}$ is the signal gain coefficient. If there is no target at $\left(\theta_{i},r_{j}\right)$, then $x_{ij}=0$. The object detection and localization is to find a set of non-zero $x_{ij}$s in Equation (11). Equation (11) can be expressed in matrix form in Figure 1. The number of targets, denoted as K, is much smaller than the number of grid points ($K\ll N_{\theta }N_{r}$). Thus, the matrix form in Figure 1 can be regarded as a CS signal model and sensing matrix A is composed of $z(t;\theta_{i},r_{j})$. The object detection searches a set of echo signals in the received signal, and indices of echo signals represent their locations.

### 2.4. Path Loss and Human Respiration Signal Model

**Figure 2:**
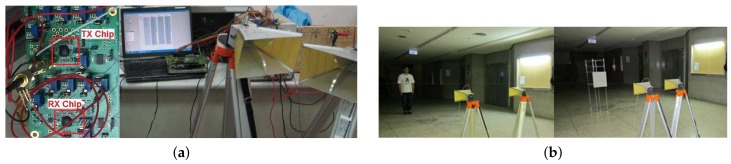
(**a**) The complementary metal-oxide-semiconductor (CMOS) radar system [10] and (**b**) measurement environments.

**Figure 3:**
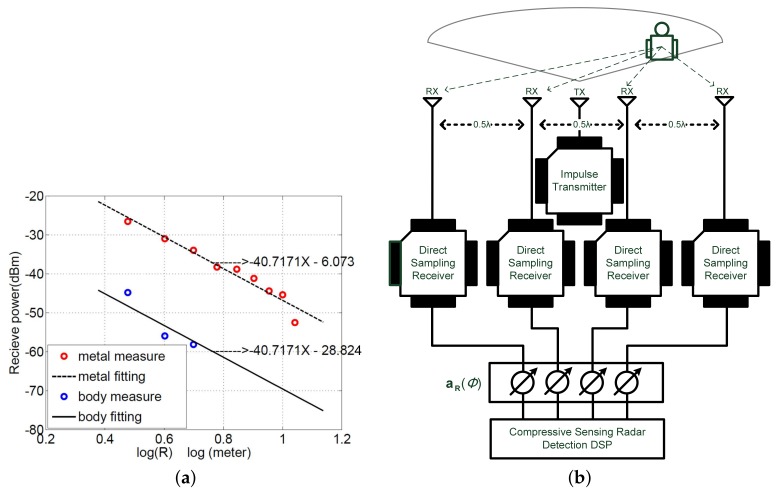
(**a**) Single-input multiple-output (SIMO) radar configuration and (**b**) the measured path loss models of the human body and metal object.

Path loss of the travelling electromagnetic wave determines the signal gain coefficient $x_{ij}$. This work utilizes a radar system [10] integrated with a CMOS impulse radar chip [1] to measure the path loss parameters of metallic objects and human bodies, as shown in Figure 2a,b. The CMOS radar system transmits 25 dBm 1 GHz sinusoidal impulse with a repetitive 10 MHz frequency, resulting in 0.94 mm range resolution. Figure 3a shows the measured results of the path loss versus logthmic radius distance. The power attenuation of human is more than 20 dB than that of a mental object due to the high energy absorption ratio of human body. The 0.94 mm resolution is fine enough to capture tiny respiratory information. This study adds four-segment linear waveform (FSLW) [10] respiration signals into the echo signals of human target to simulate the practical human respiration.

Based on the measurement results, this study constructs a SIMO impulse radar system with 4 receive antennas and 1 transmit antenna, which are configured as shown in Figure 3b. Each receive antenna receives 128 samples for each iteration. The maximum detection range is 5 m, and direction (angle) is between +45 and -45 degrees. The range resolution is $N_{r}=256$ and angle resolution is $N_{\theta }=13$, that is, target scene forms 256×13 range-azimuth grid. The sensing matrix of the CS radar system is a 512×3328 matrix. $a_{T}\left(\phi \right)$ and $a_{R}\left(\phi \right)$ are determined by (9) and (10) with $N_{t}=\;1$ and $N_{r}=4$, respectively. The receive antenna spacing $d_{R}$ is $\frac{\lambda }{2}$. There are three targets in the scene. One is a non-human object and the others are human bodies, which are modelled by the FSLW respiration model [10] with intensity A=0.01 m, inspiration speed $\beta_{1}=0.5$, expiration speed $\beta_{2}=0.5$, and respiration holding ratio X=0.5. The respiration rate of two persons are 0.5 Hz and 0.8 Hz, respectively. The non-human target is located at the distance 4.5 m and angle $22.5^{\circ }$. One person is located at the distance 2 m and angle $-7.5^{\circ }$, and the other is located at distance 3.5 m and angle $30^{\circ }$.

### 2.5. Reconstruction Algorithms for Compressive Sensing

Orthogonal matching pursuit (OMP) [26] is one of the popular greedy reconstruction algorithms for compressive sensing. For a compressive sensing framework y=Ax, y is M×1 measurement vector, A is M×N sensing matrix, and x is N×1 signal vector. First, the OMP algorithm searches the support set iteratively according to the matching results by

(12) $$\alpha =A^{T}y={\left[\begin{array}{cccc} a_{1} & a_{2} & \cdots & a_{N} \end{array}\right]}^{T}y$$

The determined index with the maximum matched gain is added into the support set $I_{k}$ of the *k*-th iteration. Then, the estimated signal vector of the *k*-th iteration, denoted as ${\hat{x}}_{k}$, can be reconstructed by ${\hat{x}}_{k}=A_{I_{k}}^{†}y$, where $A_{I_{k}}^{†}$ is the pseudo-inverse of $A_{I_{k}}$. Then, the residual of the *k*-th iteration can be calculated by $r_{k}=y-A_{I_{k}}{\hat{x}}_{k}$. The residual $r_{k}$ is then used to match the index of the next iteration by $\alpha_{k}=A_{{\tilde{I}}_{k}}^{T}r_{k}$, where ${\tilde{I}}_{k}$ is complementary set of support set $I_{k}$. Algorithm 1 shows the pseudo code of the OMP algorithm.

**Algorithm 1** Orthogonal Matching Pursuit Algorithm**Input:** Sensing matrix A, measurement y, target sparsity K **Output:** Support set $I_{k}$, estimate ${\hat{x}}_{k}$
1:Initialization:
k=1; $I_{0}=\phi$;$r_{0}=y$; $\alpha_{0}=A^{T}r_{k-1}$2:**repeat** 3:  $i_{k}=\arg\max(|\alpha_{k-1}\left|\right)$4:  $I_{k}=I_{k-1}\cup \left\{i_{k}\right\}$;5:  ${\hat{x}}_{I_{k}}=A_{I_{k}}^{†}y$;6:  $r_{k}=y-A_{I_{k}}{\hat{x}}_{I_{k}}$;7:  $\alpha_{k}=A_{{\tilde{I}}_{k}}^{T}r_{k}$8:  k=k+1;9:**until** (k≥K)



### 2.6. Orthogonal Matching Pursuit via Matrix Inversion Bypass

The computational bottleneck of the OMP algorithm is the matrix inversion in Step 5 of Algorithm 1. Thus, this study uses the Schur-Banachiewicz block-wise inversion [27,28] to reduce the complexity. The derived OMP algorithm is called the orthogonal matching pursuit via matrix inversion bypass (OMP-MIB) [29]. We denote N×N matrix $G=A^{T}A$ and $G_{I,J}=A_{I}^{T}A_{J}$, where I and J are any two support sets. Thus, the estimated signal vector can be rewritten as

(13) $${\hat{x}}_{k}=A_{I_{k}}^{†}y=G_{I_{k},I_{k}}^{-1}A_{I_{k}}^{T}y,$$

where $G_{I_{k},I_{k}}$ is a K×K matrix. Schur-Banachiewicz block-wise inversion is then applied to Equation (13). Hence, matrix inversion in Equation (13) can be expressed by

(14) $$\begin{array}{cc} G_{I_{k},I_{k}}^{-1} & ={\left[\begin{array}{cc} G_{I_{k-1},I_{k-1}} & G_{I_{k-1},i}\\ G_{i,I_{k-1}} & G_{i,i} \end{array}\right]}^{-1}\\ & =\left[\begin{array}{cc} G_{I_{k-1},I_{k-1}}^{-1}+W^{T}VW & -W^{T}V\\ -VW & V \end{array}\right] \end{array}$$

where W is $1\times \left(K-1\right)$ matrix is

(15) $$W=G_{i,I_{k-1}}G_{I_{k-1},I_{k-1}}^{-1}$$

and V is a scalar:

(16) $$V={\left(G_{i,i}-G_{i,I_{k-1}}G_{I_{k-1},I_{k-1}}^{-1}G_{I_{k-1},i}\right)}^{-1}$$

Equation (14) shows that the matrix inversion of $G_{I_{k},I_{k}}$ can be replaced by several matrix operations. Since V is a simple reciprocal operation, matrix inversion operation is not required in Equation (14). The pseudo code of the OMP-MIB algorithm is shown in Algorithm 2.

**Algorithm 2** Orthogonal Matching Pursuit via Matrix Inversion Bypass**Input:** Sensing matrix A, measurement y, target sparsity *K* **Output:** Reconstructed signal $\hat{x}$, support set $I_{k}$
1:Initialization:
$I_{0}=\phi$; $r_{0}=y$;$\alpha_{0}=A^{T}r_{0}$; $G=A^{T}A$;$i_{1}=\arg\max(|\alpha_{0}\left|\right)$, $I_{1}=I_{0}\cup \left\{i_{1}\right\}$;$G_{I_{1},I_{1}}^{-1}=1/G_{i_{1},i_{1}}$${\hat{x}}_{I_{1}}=A_{I_{1}}^{†}y$;$\alpha_{1}=A_{{\tilde{I}}_{1}}^{T}y-G_{\tilde{I_{1}},I_{1}}{\hat{x}}_{I_{1}}$;Iteration counter k=2;2:**repeat** 3:  $i_{k}=\arg\max(|\alpha_{k-1}\left|\right)$4:  $I_{k}=I_{k-1}\cup \left\{i_{k}\right\}$5:  $W=G_{i_{k},I_{k-1}}G_{I_{k-1},I_{k-1}}^{-1}$  6:  $V={\left(G_{i_{k},i_{k}}-{\mathrm{WG}}_{I_{k-1},i_{k}}\right)}^{-1}$  7:  $U=WA_{I_{k-1}}^{T}y-A_{i}^{T}y$  8:  $G_{I_{k},I_{k}}^{-1}=\left[\begin{array}{cc} G_{I_{k-1},I_{k-1}}^{-1}+{\mathrm{VW}}^{T}W & -{\mathrm{VW}}^{T}\\ -\mathrm{VW} & V \end{array}\right]$  9:  $\alpha_{k}=\alpha_{k-1}-G_{{\tilde{I}}_{k},I_{k}}\left[\begin{array}{c} {\mathrm{VUW}}^{T}\\ -\mathrm{VU} \end{array}\right]$  10:  ${\hat{x}}_{k}=\left[\begin{array}{c} {\hat{x}}_{k-1}\\ 0 \end{array}\right]+\left[\begin{array}{c} W^{T}VU\\ -VU \end{array}\right]$  11:**until** k≥K



## 3. Two-Stage Reconstruction Algorithm

**Figure 4:**
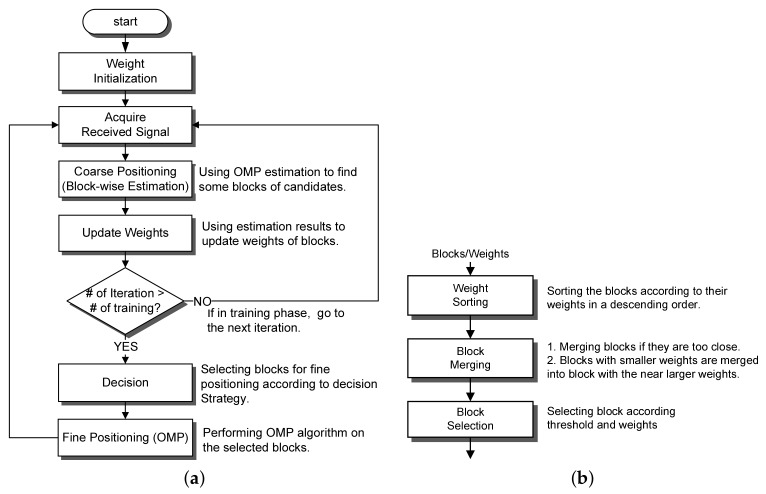
(**a**) Flow chart of the proposed low-complexity two-stage OMP reconstruction algorithm and (**b**) the decision strategy.

The complexity of the OMP reconstruction algorithm increases along with the increased sensing resolution and dimension of the sensing matrix. In order to reduce high reconstruction complexity for the CMOS impulse radar system, this study proposes a two-stage OMP reconstruction algorithm including block-wise OMP estimation, weight updating and decision mechanism, and fine estimation. Figure 4a shows the processing flow chart of the proposed algorithm.

### 3.1. Block-Wise OMP Estimation

The proposed algorithm first performs block-wise OMP, which reduces the sensing matrix size to estimate the rough target regions. In the CS radar, each column of a sensing matrix A represents the echo signal of a specific grid point. Because the neighbouring grid points have similar echo signals, A can be separated into several blocks and each block is the concatenation of neighbouring columns. Thus, the target locations can be coarsely estimated by the block-wise OMP estimation. The block-wise OMP algorithm first downsizes A into a new sensing matrix D by $D_{j}=A_{\left(j-1\right)\times \left(B+1\right)}+\cdots +A_{j\times B}$, where *B* is the block size. The *j*-th column of a downsized sensing matrix D is the sum of columns in the *j*-th block of A. The block-wise OMP estimation can be regarded as the OMP using D as the sensing matrix. The detailed block-wise OMP algorithm is shown in Algorithm 3. The block-wise OMP estimation selects several block candidates and stores their indices in a candidate set $I_{block}$ for the following weight updating.

**Algorithm 3** Block-Wise Estimation Algorithm**Input:** Sensing matrix A, measurement y, target sparsity K, block size B **Output:** Support set $I_{k}$
1:Initialization:
*k=1;* $I_{0}=\phi$;New sensing matrix D where $D_{j}=A_{\left(j-1\right)B+1}+\cdots +A_{jB}$*$r_{0}=y$; $\alpha_{0}=D^{T}r_{k-1}$*
2:**repeat** 3:  $i_{\mathrm{block}}=\arg\max(|\alpha_{k-1}\left|\right)$4:  $I_{k}=I_{k-1}\cup \left\{i_{\mathrm{block}}\right\}$;5:  ${\hat{x}}_{I_{k}}=D_{I_{k}}^{†}y$;6:  $r_{k}=y-D_{I_{k}}{\hat{x}}_{I_{k}}$;7:  $\alpha_{k}=D_{{\tilde{I}}_{k}}^{T}r_{k}$8:  *k=k+1;*9:***until*** (k≥K)


### 3.2. Weight Updating

Each block has a weight representing the possibility of having targets within the block. The proposed algorithm calculates weights by using historical coarse estimation results. The proposed weighting mechanism utilizes the fact that the blocks near a known object have higher opportunities of having objects in, even in a noisy environment. Thus, the proposed algorithm collects coarse estimation results of the previous iteration for evaluating the block weights. The weight updating is expressed by $Z\left(I_{block}\right)=Z\left(I_{block}\right)+1$, where is the candidate set selected by the coarse estimation. The size of $I_{block}$ is determined by the number of block candidates $K_{b}$ of the compressive sensing system. The algorithm adopts several sensing matrix A from the UWB radar system and skips the fine estimation in the first a few $T_{num}$ iterations of the training mode. This weight computation helps to increase the reliability of the weight distribution. The algorithm usually needs only one or two training iterations in low-noise conditions.

### 3.3. Decision Strategy for Fine Estimation

After updating weights, the algorithm selects the blocks to be performed by the following fine estimation according to the decision strategy shown in Figure 4b. The first step performs weight sorting. The blocks are sorted in the descending order of their weights. The second step is block merging. Since the size of human body is much larger than the respiratory spatial variation, the neighbouring selected blocks could represent the same target. Thus, this step merges the block candidates within a merging distance $M_{g}$ into the block with the largest weight. Then, the merged blocks are discarded and their weights are added to the weight of the winner block. The final step selects the remaining candidate blocks by comparing the normalized weights with a specified threshold $T_{h}$. After the threshold decision, the fine estimation combines the columns of the selected blocks as sensing matrix to perform the fine object positioning. Notice that the block merging in the decision process assumes that the size of human body is much larger than the respiratory spatial variation because the radar ranging resolution is much higher than the body resolution. Thus, sparsity K in the fine estimation OMP is no larger than $K_{b}$ to locate the detailed positions of the human bodies or objects. Following simulation and hardware design assume *K* = $K_{b}$ without the loss of generality.

After the location of targets are estimated by the OMP estimation, the algorithm returns back to the next iteration. Notice that the block weights are not reset and remain the same at the beginning of the next iteration. The detailed two-stage reconstruction algorithm is shown in Algorithm 4.

**Algorithm 4** Proposed Two-Stage Reconstruction Algorithm**Input:** sensing matrix A; received signal y; number of targets K; block size B; training number $T_{\mathrm{num}}$; threshold $T_{h}$; merging distance $M_{g}$; number of block candidates $K_{b}$; **Output:** Support set $I_{out}$
1:Initialization: Let iteration counter k=1, and initialize weights $Z\left(1:\frac{N}{B}\right)=0$;2:Acquire received signal $y_{k}$ of *k*-th iteration;3:Coarse positioning(Block-Wise Estimation Algorithm):(A)Calculate downsized sensing matrix D where $D_{j}=A_{\left(j-1\right)B+1}+\cdots +A_{jB}$(B)$I_{block}=OMP\left(D,y_{k},K_{b}\right)$;4:Update weights: $Z\left(I_{block}\right)=Z\left(I_{block}\right)+1$;5:**if** $k\leq T_{num}$ **then**6:  Let k=k+1 and goto Step 2 for next iteration;7:**end** **if**8:Decision:
(A)Sorting: $\left[Z_{sort},I_{sort}\right]=Sort\left(Z\left(I_{block}\right),I_{block}\right)$(B)Merging:
**for all** *i* and *j* such that $|I_{sort}\left(i\right)-I_{sort}\left(j\right)|<M_{g}\;\mathrm{and}\;i\neq j$ **do**  **if** $Z_{sort}\left(i\right)>Z_{sort}\left(j\right)$ **then**   $Z_{sort}\left(i\right)=Z_{sort}\left(i\right)+Z_{sort}\left(j\right)$   and remove $I_{sort}\left(j\right)$ from $I_{sort}$  **else**   $Z_{sort}\left(j\right)=Z_{sort}\left(j\right)+Z_{sort}\left(i\right)$   and remove $I_{sort}\left(i\right)$ from $I_{sort}$  **end if****end** **for**(C)Selection:
Threshold calculation: $t=T_{h}\times sum\left(Z\right)$$I_{select}=I_{sort}\left(1:K\right)$
Removing $I_{select}\left(i\right)$ from $I_{select}$ for every *i* that satisfies $Z_{sort}\left(i\right)<t$9:  Fine positioning: $I_{out}=OMP\left(A_{I_{select}},y_{k},K\right)$;10:  Output $I_{out}$, and let k=k+1;11:  Goto Step 2 in order to acquire new received signal;


## 4. Complexity and Performance Analysis

This section analyses the computational complexity of the conventional OMP algorithm, OMP via MIB algorithm, and the proposed two-stage OMP algorithms with and without MIB. The complexity analysis is based on the number of multiplications of several matrix operations. The complexity of the matrix inversion for an n×n matrix is $O(n^{3})$. The complexity of the matrix multiplication A×B is O(mnk), where A is an m×n matrix and B is an n×k matrix. The complexity of the matrix pseudo-inverse $A^{†}$ is $O(n^{3}+2mn^{2})$.

### 4.1. Orthogonal Matching Pursuit Algorithm

Algorithm 1 shows the OMP algorithm. The computational complexity of the pseudo matrix inversion and multiplication in Step 5 is $O(k^{3}+2mk^{2}+mk)$. The complexity of Step 6 and Step 7 are O(mk) and O((n-k)m). Thus, total complexity of the OMP algorithm is $O(k^{3}+2mk^{2}+nm+mk)$. For the fair comparison with the OMP-MIB algorithm, the computation of matrix pseudo-inverse $A_{I_{k}}^{†}={\left(A_{I_{k}}^{T}A_{I_{k}}\right)}^{-1}A_{I_{k}}^{T}$ can be reduced to $A_{I_{k}}^{†}={\left(G_{I_{k},I_{k}}\right)}^{-1}A_{I_{k}}^{T}$. Therefore, computational complexity of matrix pseudo-inverse $A_{I_{k}}^{†}$ is $O(k^{3}+mk^{2})$. Thus, total complexity of the OMP algorithm is $O(k^{3}+mk^{2}+nm+mk)$.

### 4.2. OMP-MIB Algorithm

Algorithm 2 shows the OMP-MIB algorithm. The complexity of calculating W,V, and U are $O({\left(k-1\right)}^{2})$, O(k), and O(mk+k-1), respectively. Steps 8, 9, and 10 calculate $G_{I_{k},I_{k}}^{-1},\alpha_{k},and\;{\hat{x}}_{k}$, respectively. The computational complexity of calculating $VW^{T}W$ in Step 8 is $O({\left(k-1\right)}^{2}+\left(k-1\right))$. In Step 9, the complexity of ${\left[\begin{array}{cc} {\mathrm{VUW}}^{T} & -\mathrm{VU} \end{array}\right]}^{T}$ is O(k). Thus, computational complexity of calculating $G_{\tilde{I_{k}},I_{k}}\left[\begin{array}{c} {\mathrm{VUW}}^{T}\\ -\mathrm{VU} \end{array}\right]$ is $O(nk-k^{2})$. Thus, the total complexity of the OMP via MIB algorithm is $O\left(k^{2}+nk+mk-1\right)\approx O\left(k^{2}+nk+mk\right)$.

### 4.3. Proposed Two-Stage OMP Reconstruction Algorithm

The major complexity of the proposed two-stage reconstruction algorithm are the OMP calculations for both coarse and fine positioning. The block-wise estimation is an OMP algorithm for a downsized $m\times n_{c}$ sensing matrix D. Thus, the complexity of the coarse positioning is $O\left(k^{3}+mk^{2}+n_{c}m+mk\right)$. For block size *b*, $n_{c}$ can be expressed as $n_{c}=\frac{n}{b}$. Thus, the complexity of the coarse positioning becomes $O\left(k^{3}+mk^{2}+\frac{n}{b}m+mk\right)$. The fine positioning uses a $m\times n_{f}$ sensing matrix to perform the OMP algorithm for the selected blocks. Thus, the complexity is $O\left(k^{3}+mk^{2}+n_{f}m+mk\right)$, and $n_{f}$ can be expressed as $n_{f}=kb$. Hence, the complexity of the fine positioning becomes $O\left(k^{3}+mk^{2}+kbm+mk\right)$. The total complexity of the proposed two-stage reconstruction algorithm is $O\left(k^{3}+mk^{2}+\frac{n}{b}m+mk\right)+O\left(k^{3}+mk^{2}+kbm+mk\right)$.

In addition, both the coarse and fine positioning can be realized by using OMP-MIB algorithm. Thus, the complexity of coarse and fine positioning are $O\left(k^{2}+n_{c}k+mk\right)$ and $O\left(k^{2}+n_{f}k+mk\right)$, respectively. By substituting $n_{c}=\frac{n}{b}$ and $n_{f}=kb$ into $O\left(k^{2}+n_{c}k+mk\right)$ and $O\left(k^{2}+n_{f}k+mk\right)$, respectively, Thus, the complexity of the coarse and fine positioning by using OMP-MIB algorithm are $O\left(k^{2}+\frac{n}{b}k+mk\right)$ and $O\left(\left(b+1\right)k^{2}+mk\right)$.

**Figure 5:**
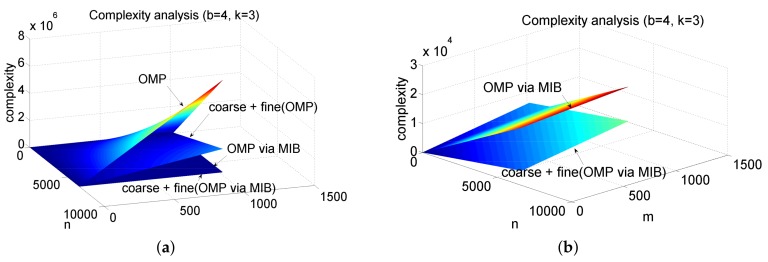
Complexity analysis results of (**a**) the orthogonal matching pursuit (OMP) and two-stage OMP algorithms w/o OMP-Matrix Inversion Bypass (MIB) scheme and (**b**) the OMP and two-stage OMP algorithms with OMP-MIB scheme.

Figure 5a shows the complexity analysis results with the sparsity factor k=3 and block size b=4. The complexity of two-stage OMP algorithm is much lower than the OMP algorithm. The OMP via MIB algorithm can further reduce the complexity of the OMP and two-stage OMP algorithms, as shown in Figure 5b. In this case, the proposed two-stage algorithm still has lower complexity.

**Figure 6:**
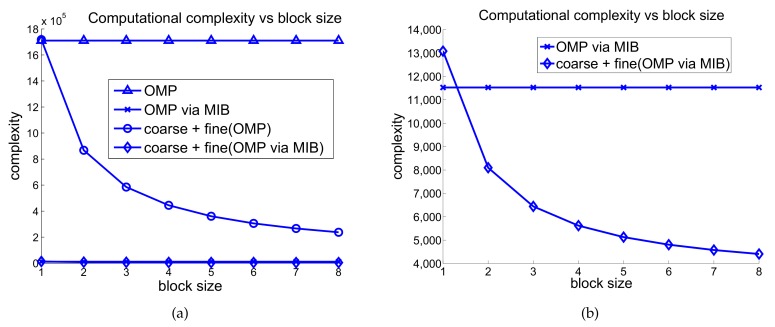
Computational complexity versus block size where m=512, and n=3328.

Figure 6 shows the complexity of various block sizes. Figure 6b shows the complexity of the OMP-MIB and the proposed two-stage OMP-MIB algorithm since they are too close in Figure 6a. The complexity of the proposed algorithm decreases along with the increased block size because of the decreased dimension of the sensing matrix of the block-wise estimation. The complexity of the proposed two-stage OMP reconstruction with block size b=4 reduces approximately 75% complexity of the conventional OMP algorithm. Figure 6b shows that proposed two-stage OMP-MIB algorithm with block size b=4 reduces approximately 50% complexity comparing to original OMP via MIB algorithm. The complexity reduction percentage would further increases when the radar resolution increases, that is, the dimension of the original sensing matrix A increases. This is a great benefit to human detection because the impulse radar usually requires very high radio frequency and very high spatial resolution in order to acquire the tiny movement of the human respiration.

### 4.4. Simulation Result

**Figure 7:**
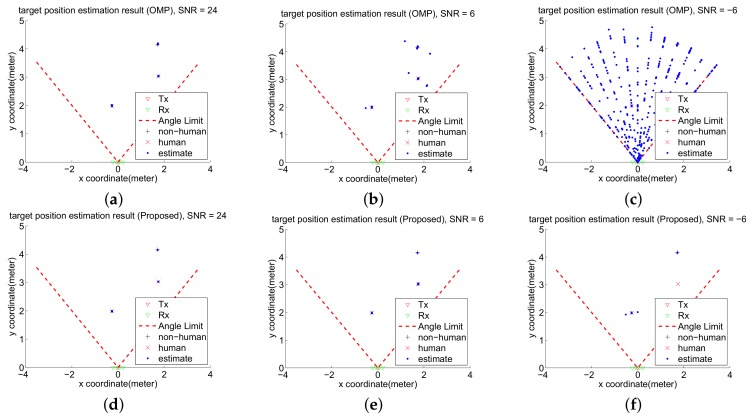
The compressive sensing (CS) radar scenes using the OMP reconstruction in (**a**) 24 dB, (**b**) 6 dB, and (**c**) −6 dB SNR environments. The CS radar scenes using the two-stage OMP reconstruction in (**d**) 24 dB, (**e**) 6 dB, and (**f**) −6 dB SNR environments.

Figure 7 shows the positioning results of the OMP and two-stage OMP algorithms. 500 impulses are used to simulate the CS radar processing under different SNR conditions. The sensing matrix **A** is derived from the MIMO compressive sensing model in Section 2.2–Section 2.4. The “estimate” points are the detected targets, which are sent to the respiration feature extraction algorithm [10] to identify the human and object. When SNR is 24 dB (Figure 7a,d), both the OMP and two-stage OMP algorithm can perfectly locate the object and humans. When SNR is 6 dB (Figure 7b,e), the OMP algorithm generates a few incorrect estimation results of the object and human locations, but the proposed algorithm still perfectly locates the object and humans. When SNR is −6 dB (Figure 7c,f). However, the proposed algorithm can still detect one human target with some incorrect results and detect the non-human target perfectly. The proposed two-stage reconstruction algorithm has better positioning performance than the conventional OMP algorithm especially in noisy environments.

### 4.5. Performance Analysis

This study uses three metrics to perform detailed analysis of the reconstruction and detection results including normalized mean square error (NMSE), number of hits, and hit ratio. These metrics show some properties that are not revealed in Figure 7.

The normalized mean squared error of the reconstructed signals by CS radar is defined by

(17) $$\mathrm{NMSE}=\frac{{∥y_{estimate}-y_{true}∥}_{2}^{2}}{{∥y_{true}∥}_{2}^{2}},$$

where $y_{true}$ is the received radar signal and $y_{estimate}$ is the reconstructed signal by the OMP or the proposed algorithm. Number of hits is the number of correct estimates in the simulation. The hit ratio is the detection probability defined by

(18) $$\mathrm{Hit}\;\mathrm{Ratio}=\frac{\mathrm{Number}\;\mathrm{of}\;\mathrm{Correct}\;\mathrm{Estimates}}{\mathrm{Number}\;\mathrm{of}\;\mathrm{Total}\;\mathrm{Estimates}}.$$

**Figure 8:**
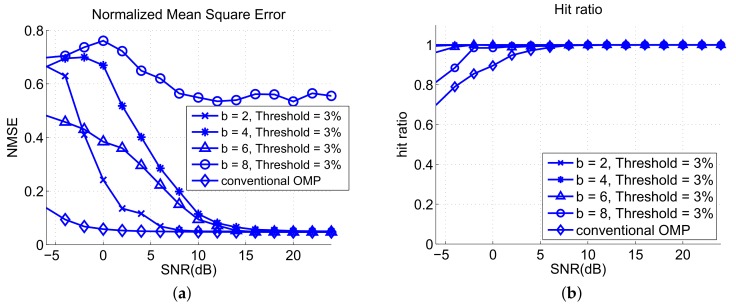
Analysis results of (**a**) NMSE and (**b**) hit ratio for different block sizes.

Figure 8a shows the NMSE verus SNR performances of the OMP and two-stage OMP algorithms with different block sizes. The proposed two-stage OMP algorithm has worse NMSE performance because the proposed algorithm utilizes threshold mechanism to discard bad estimates and distort the reconstruction signal. However, the NMSE does not necessarily reflect the detection and positioning performance, as shown in Figure 7, because a lower NMSE value only means that the signal is better reconstructed by the OMP process but the reconstructed signal has an incorrect combination of indices, that is, the support set $I_{K}$. The proposed algorithm with b=6 has better NMSE than that with b=4 because the threshold mechanism with b=4 is more strict than that with b=6 because the block weights is not normalized as we mentioned in Section 3. Hence, the proposed algorithm with b=6 makes estimated results more easily to reach the threshold, leading to lower NMSE. This is similar to the reason that causes the better RMSE of the OMP algorithm. Figure 8b shows that the hit ratio of the two-stage OMP is better than that of the OMP. This implies that the matching pursuit quality of the two-stage OMP algorithm is better than that of the OMP algorithm.

**Figure 9:**
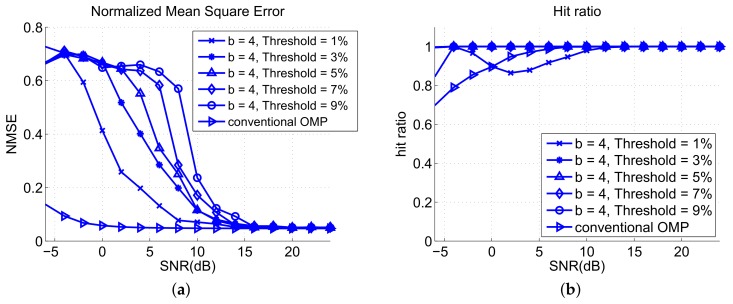
Analysis results of (**a**) normalized mean square error (NMSE) and (**b**) hit ratio for different thresholds.

Figure 9a shows the NMSE versus SNR with different thresholds. The OMP has lower NMSE than the proposed algorithm because of the threshold mechanism. A higher threshold of the proposed algorithm causes a higher NMSE value because more matched results are discarded. Figure 9b shows the hit ratio of the algorithms for different thresholds. A lower threshold leads to a larger number of hits. Threshold $T_{h}=1\%$ has the highest hit number in the analysis of the proposed algorithm, but the hit ratio is unstable when SNR lower than 10 dB.

## 5. Architecture Design and Implementation

This section introduces the hardware design and implementation of the proposed two-stage reconstruction algorithm. The OMP processing for coarse and fine positioning are the most complex and time consuming steps. Thus, an OMP processor was designed to accelerate the reconstruction processing.

### 5.1. Architecture of OMP via MIB Processor

**Figure 10:**
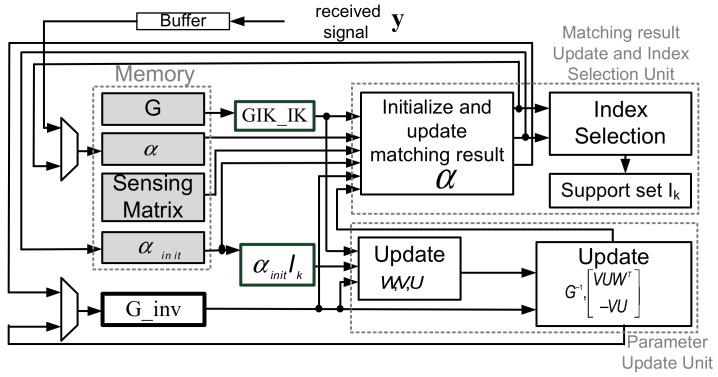
Block diagram of the proposed architecture.

Figure 10 shows the overall architecture, which can be divided into two parts. One is Matching Result Update and Index Selection Unit, and the other is Parameter Update Unit. The gray blocks are data memories including ROMs for *G* and sensing matrix, a single-port SRAM for $\alpha_{init}$, and a dual-port SRAM for α. G_inv, GIK_IK, $\alpha_{init}I_{k}$, and support set $I_{k}$ are register file. GIK_IK and $\alpha_{init}I_{k}$ are buffers for parallel data streams. $I_{k}$ is the storage of support set. Initially, α serves as register and loads the received signal firstly for calculating matching result, and then initialization steps are executed by matching result update circuit and index selection circuit. After initialization, updating GIK_IK and $\alpha_{init}I_{k}$ that store specific value from G ROM and $\alpha_{init}$ RAM for computing parameter W, V, U, and W, V, U are calculated by parameter update unit sequentially. Then $G_{I_{k},I_{k}}^{-1}$ and $\alpha_{k}$ are updated. Support set is also updated during the process of updating $\alpha_{k}$. Then the algorithm goes to next iteration.

### 5.2. Matching Result Update and Index Selection Unit

#### 5.2.1. Initialization and Matching Result Update Circuit

**Figure 11:**
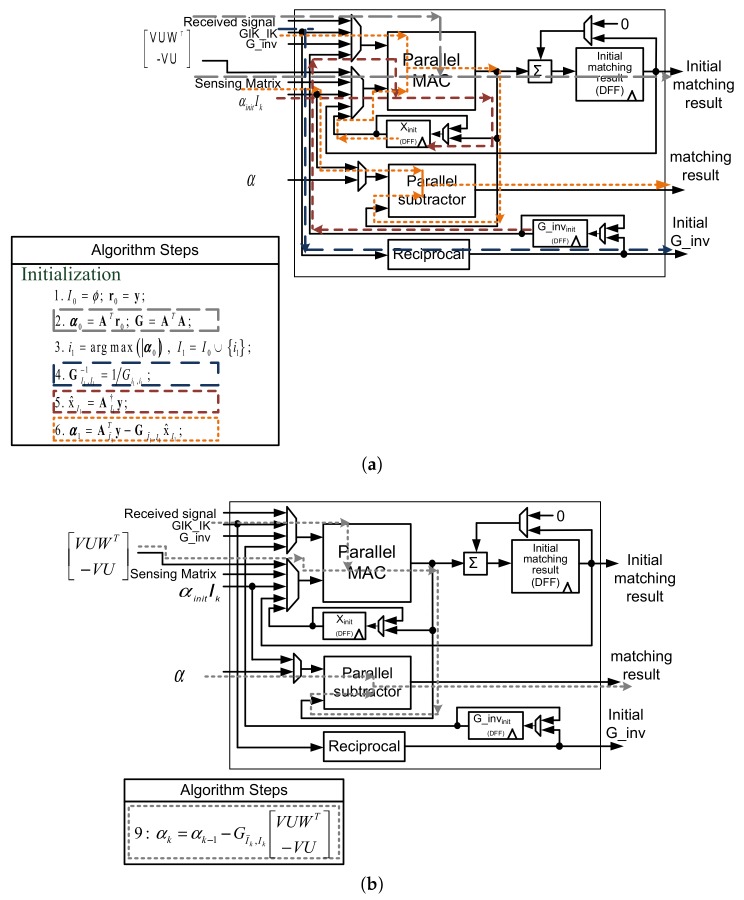
Processing flows of matching result update circuit for (**a**) initialization and (**b**) common matching result update in the data path circuits.

Figure 11 shows the matching result update circuit. The Parallel MAC consists of $N_{\mathrm{mul}}$ multiply-and-accumulate circuits (MACs) that determine the speed of matrix multiplication. The number of subtractor circuits in the Parallel Subtractor is also $N_{\mathrm{mul}}$.

The matching result update circuit not only calculates the matching result but also performs the initialization of the OMP-MIB algorithm. In initialization phase, parallel MAC calculates the initial matching result first. Because of the extremely high dimension of sensing matrix and received signals, it is impossible to execute the parallel MAC for vector-vector multiplication of each column of sensing matrix in a clock cycle. Thus, the partial results are accumulated by summation circuit and registers. For an M×N sensing matrix, the matching result update circuit produces one element of $\alpha_{0}$ in every $\frac{M}{N_{\mathrm{mul}}}$ clock cycles. Since the elements of initial matching result vector $\alpha_{0}$ are computed sequentially, index selection circuit compares the maximum value of initial matching result vector one by one, therefore, reducing the complexity of index selection circuit. The resultant $\alpha_{0}$ is then stored in memory $\alpha_{init}$. The calculations of initial matching result can be finished in $N\frac{M}{N_{\mathrm{mul}}}$ clock cycles.

In the initialization phase, index $i_{1}$ is computed by index selection circuit, and $G_{I_{1},I_{1}}^{-1}$ is computed by reciprocal circuit. After $G_{I_{1},I_{1}}^{-1}$ is calculated, ${\hat{x}}_{I_{1}}$ is produced by parallel MAC and stored in $x_{init}$, which will be used for the calculation of new matching result $\alpha_{1}$ by parallel MAC and parallel subtractor circuits. Figure 11a shows the processing flow of the initialization in the data path circuit.

The matching result update circuit updates $\alpha_{k}$ based on $\alpha_{k-1}$ and matrices W, V, U. Dual-port SRAM is used for simultaneous reading and writing of αs. $\alpha_{k}$ is obtained by subtracting $\alpha_{k-1}$ by the parallel MAC output of the matrix multiplication $G_{{\tilde{I}}_{k},I_{k}}\times \left[\begin{array}{c} {\mathrm{VUW}}^{T}\\ -\mathrm{VU} \end{array}\right]$. Parallel MAC calculates $N_{\mathrm{mul}}$ elements of matrix multiplication operation in every *k* clock cycles, where *k* is the iteration counter and $N_{\mathrm{mul}}$ is the number of MAC circuits. The $N_{\mathrm{mul}}$ partial results from parallel MAC subtract the matching result vector $\alpha_{k-1}$ concurrently by parallel subtractor circuits to obtain $N_{\mathrm{mul}}$ elements of $\alpha_{k}$. There are N-k elements in the vector $\alpha_{k}$ for a M×N sensing matrix and $N-k>>N_{\mathrm{mul}}$. Therefore, the matching result updating is the most time consuming step in the algorithm. Figure 11b shows the processing flow of matching result updating process.

#### 5.2.2. Index Selection Circuit

**Figure 12:**
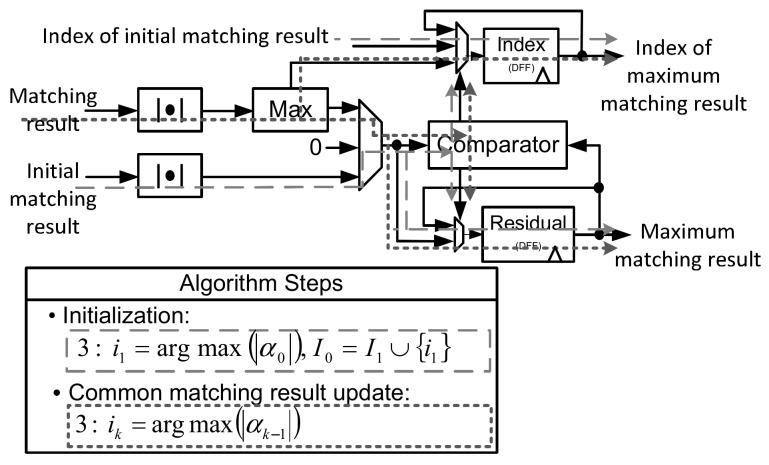
Data path of index selection circuit.

Figure 12 shows the index selection circuit. At initialization stage, the matching result update circuit calculates the elements of initial matching result vector $\alpha_{0}$ sequentially, and then index selection circuit compares and selects the index of the elements with maximum matching result. At the iteration stage, the matching result update circuit outputs $N_{\mathrm{mul}}$ matching results concurrently. Therefore, index selection circuit compares these $N_{\mathrm{mul}}$ matching results by using maximum circuit. Maximum matching result of the maximum circuit is further compared with the previous maximum matching result. The larger result is preserved for the next comparison. Figure 12 shows processing flows of index selection circuit for the initialization and common matching result update processes.

### 5.3. Parameter Update Unit

**Figure 13:**
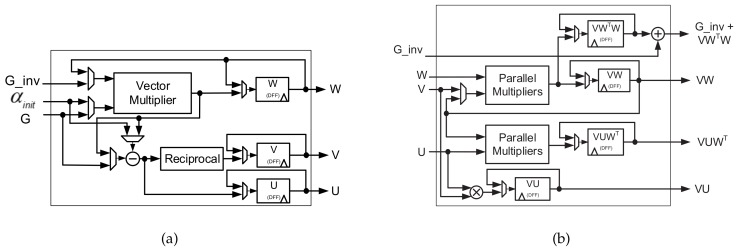
Architecture of parameter update unit where (**a**) updates W, V, U and (**b**) updates G_inv, ${\mathrm{VUW}}^{T}$, VU.

Figure 13 shows the architecture of parameter update unit, which consists two circuits to update the matrices W, V, U. The vector multiplier and parallel multiplier both use K-1 multipliers to execute vector multiplication and parallel multiplication. Figure 13a shows the parameter update circuit for W, V, and U. W is first calculated in k-1 clock cycles in the *k*-th iteration. Then, V and U are computed in one clock cycle. After W, V and U are updated, the circuit shown in Figure 13b computes the matrix multiplications of VW, ${\mathrm{VUW}}^{T}$, and ${\mathrm{VW}}^{T}W$, which are used for updating new matching result $\alpha_{k}$ and $G_{{\tilde{I}}_{k},I_{k}}^{-1}$ stored in memory G_inv.

Figure 13b shows the several matrix multiplication circuits. First, VW is calculated in one clock cycle. Then, VU, ${\mathrm{VUW}}^{T}$, and ${\mathrm{VW}}^{T}W$ are calculated concurrently. VU and ${\mathrm{VUW}}^{T}$ require one clock cycle, and ${\mathrm{VW}}^{T}W$ requires k-1 clock cycles. After these matrices are updated, new matching result $\alpha_{k}$ and index $i_{k}$ can be determined by the matching result update circuit and index selection circuit.

In the block diagrams of the afore-mentioned circuits, some processing modules, such as parallel MACs, parallel substractors, parallel multipliers, and vector multiplier, can be referred to our previous works [30,31].

### 5.4. Implementation Results and Comparison

The proposed two-stage OMP processor was designed and implemented by using Software and Xilinx Virtex-7 FPGA (Xilinx, San Jose, CA, USA). The proposed two-stage reconstruction algorithm was realized by Matlab software (MathWorks, Natick, MA, USA) except that the pure OMP processing functions (Line 3.(B) and 9 in Algorithm 4) are accelerated by FPGA hardware. The original CS radar system has a 512×3328 sensing matrix derived from the MIMO compressive sensing model in Section III B, C, and D, with b=4, k=8, and SNR = −20 dB, that is, the coarse estimation performs OMP reconstruction for a 512×832 sensing matrix, and the fine estimation performs OMP reconstruction for a 512×32 sensing matrix. The OMP processor was implemented for both 512×832 and 512×32 sensing matrices. In the fixed-point simulation, the number of correctly selected indices in $I_{k}$ are used to determine the number of fractional bits with 13 integer bits of the signals in memory.

Table 1 shows the hardware resources utilized in the proposed processor. The processing time for reconstruction of a 512×3328 CS matrix is 35 ms. The corresponding radar image frame rate is about 28.2 frames per second, which approaches the typical video frame rate. Table 2 compares the proposed OMP processor with other reconstruction processors in the literature. The major differences of the proposed processor are the large compressing sensing matrix size and targeted application. The CS radar system has an extremely large CS matrix dimension to provide very high radar image resolution for the detection of very fine human respiration signal. Thus, it is difficult and almost impossible to implement such a high-dimension signal reconstruction processor for the CS radar system. The proposed two-stage reconstruction algorithm successfully reduces the complexity by reducing the CS matrix dimension in the coarse positioning and still maintains the high-precision reconstruction by fine-positioning. Although traditional reconstruction processors have small processing latencies, they only support much lower dimensions than the proposed two-stage OMP processor. If these traditional algorithms are applied to the compressive sensing radar system with such a high dimension, it is impossible to realize a real-time processor for the CS radar system. In the application aspects, the proposed processor supports 28.2 frames per second for the radar image real-time display. This image refreshing throughput is good enough for the human vision response.

## 6. Conclusions

This study proposes a two-stage reconstruction algorithm for the compressive sensing radar. The proposed algorithm has better positioning performance and lower complexity than the traditional OMP algorithm. The OMP can be replaced by any reduced-complexity algorithm, such as OMP via MIB, but the proposed two-stage reconstruction is still effective in reducing cost and improving performance. This work applied a practically-measured path-loss model and a human respiratory signal model from a CMOS impulse radar system to configure a 4 × 1 SIMO radar system to analyse the proposed algorithm. The proposed algorithm utilizes block-wise OMP estimation, weight threshold mechanism, and fine estimation to reduce the complexity and improve the performance. The simulation results show that the proposed algorithm needs only 25% computational complexity of the conventional OMP algorithm and has a better positioning performance than the OMP algorithm especially in noisy environments.

## Figures and Tables

**Table 1:** FPGA Implementation Results.

Plate-Form	Xlinx FPGA + Software
**Model**	**Vertex-7**
Slice Registers	6535
Slice LUTs	207,967
Block RAMs	1092
DSPs	560
Clock	318 MHz
Latency	35.4 ms
Radar Image Resolution	256×13
Radar Image Rate	28.2 frames/s

**Table 2:** VLSI implementations of OMP processors.

	Proposed	[19]	[21]	[29]	[32]	[30]	[33]
Algorithm	Two-Stage OMB-MIB	OMP	OMP	OMP-MIB	OMP	PIS-MIB-SOMP	SGP
Technology	Virtex-7	Virtex-5	65 nm	65 nm	Vertex-6	90 nm	90 nm
(N,M)	(3328,512)	(128,32)	(256,64)	(256,150)	(1024,256)	(1024,256)	(256,64)
Sparsity	8	5	8	variable	36	12	8
Clock (MHz)	318	39	165	500	120	141	150
Latency Time (μs)	35,400	24	13.7	NONE	340	72.2	61.97
Function	Radar Object Detection	Signal Reconstruct	Signal Reconstruct	Signal Reconstruct	Signal Reconstruct	Signal Reconstruct	Signal Reconstruct
